# Overlapping community detection based on bridging structural features and fuzzy C-means

**DOI:** 10.1371/journal.pone.0328825

**Published:** 2025-08-26

**Authors:** Ailian Wang, Mingwu Li, Xuyang Gao, Bolin Li, Zhiqiang Su

**Affiliations:** College of Computer Science and Technology (College of Data Science), Taiyuan University of Technology, Taiyuan, China; University of Delhi, INDIA

## Abstract

In recent years, research on community structure for complex networks has received increasing greater attention, and the overlapping community structure is more closely related to the actual social structure than the non-overlapping community structure, so it is necessary to identify and detect the overlapping communities of social networks. In this paper, we propose an overlapping community optimization method (OSFCM) based on network structure characteristics and fuzzy C-means clustering. We first abstract the feature vector matrix of each node from the network structural properties, and then optimize this matrix by a new objective function gradient optimization method, we generate the preliminary community delineation results with FCM method, and finally calibrate the communities to which the nodes belong. Experimental results show that the algorithm exhibits higher delineation accuracy and better algorithmic performance on seven real network datasets and four synthetic networks.

## Introduction

In recent years, with the popularization of the Internet and widespread use of mobile devices, social networks play an increasingly important role in people’s lives, work, and society. Simultaneously, with growing user bases and network coverage, the scale of social networks is increasing, and the structure is becoming more and more complex. In general, social networks are characterized by many complex network features, including but not limited to the small-world phenomenon [1], scale-free [2], and community structure [3]. A community represents a collection of nodes with common characteristics in the network, and community segmentation involves analyzing network structure information to discover potential communities in social networks, in order to reveal the group structure and connection characteristics between nodes in the network, so as to help understand the organization and behavioral patterns of social networks [4]. Based on the unique characteristics of “community” in social networks, community discovery algorithms have been widely used in business, politics, academia, healthcare and other fields [5,6].

Formal conceptual and algorithmic research on community segmentation began with the GN algorithm proposed by Girvan and Newman in 2001 [3], and community segmentation algorithms have gained more and more attention from scholars in the following two decades [7–9]. However, many scholars’ work focuses on the detection of non-overlapping communities where network nodes belong to only a single community, such as the CNM algorithm [10], Louvain’s algorithm [11], spectral clustering optimization algorithms, faction filtering algorithms [12], label propagation algorithms [13], and a series of global or local optimization algorithms, such as local edge clustering optimization algorithms [14]. In fact, for most social networks, nodes in the network have multiple community labels, which may simultaneously belong to different communities, e.g., in an interest network, an individual is able to have multiple interests at the same time; in a collaborative network, a writer can work with writers from multiple different fields. Therefore, network detection of overlapping communities can further reveal the complex inner organizational structure and patterns of the social network.

So far, researchers have proposed a variety of overlapping community detection methods by applying different approaches [15–17], and these methods are classified into four categories: based on Clique Percolation Method, based on label propagation, based on local expansion and optimization, based on link division and based on Machine learning. However, these methods are often plagued by certain limitations, such as most of the label optimization algorithms tend to consider only the local information of the nodes and ignore the global importance of the nodes, or there are problems such as inaccurate accuracy of community division and unsatisfactory experimental results. Therefore, we investigate the idea of fuzzy clustering and proposes a fuzzy C-means clustering overlapping community detection method based on the structural characteristics of the network. We first abstract the structural characteristics of each node in the network, use the structural characteristics to construct the feature vector matrix in fuzzy clustering, and then propose a new objective function gradient optimization method to optimize the feature vector matrix, so that the value of the difference of the eigenvectors of the similar nodes becomes smaller, and the value of the difference of the eigenvectors of the non-similar nodes becomes larger, and then according to the eigenvector matrix and the fuzzy C-means clustering analysis method, we generate the preliminary community delineation results, and finally calibrate the community label to which each node belongs to determine the final community to which each node belongs. The main contributions of this paper are as follows:

- We propose an optimized fuzzy C-means method for overlapping community detection, which solves the problem that nodes can only belong to a single community in the classical community detection algorithm, and adapts to the detection needs of overlapping communities.
- We introduce a novel objective function and gradient optimization algorithm that can effectively reduce the differences between feature vectors of similar nodes while increasing the differences between those of dissimilar nodes, allowing the structural properties of the network to potentially represent community characteristics.
- We propose a new community calibration approach to optimize the community detection results by calibrating the community to which a node belongs with the influence of the node’s neighbors both inside and outside the community on the node.
- We validate the stability and superiority of our algorithm on real network datasets and synthetic networks by simulating multi-experiments, and verify that it has higher accuracy and better performance than other methods in terms of NMI and modularity.

The rest of this paper is organized as follows. The relate work section reviews related work and introduces the current research status of overlapping community detection algorithms. The solution for OSFCM section describes the main ideas and detailed processes of our proposed algorithm. The experimental results and analysis section presents the validation process of the algorithm, including evaluation metrics, test datasets, and comparative experimental results. Finally, the conclusion section provides conclusions and perspectives.

## Relate work

This chapter introduces some traditional overlapping community detection methods and their optimization algorithms, and briefly describes the advantages and disadvantages of these algorithms.

**Based on Clique Percolation Method (CPM)**. CPM algorithm [18] is a widely used overlapping community detection method, the core of this algorithm is to decompose all fully-connected subgraphs (k-clique) from the network and perform community diffusion through overlapping nodes between different subgraphs (if any), and then identify the community information in the network based on the associations between these subgraphs. CPMd algorithm [19] is an application of CPM algorithm to directed graphs, in which a new k-clique is defined, but its core is still to find fully connected subgraphs in the network and then perform community diffusion. CBLA algorithm [20] is a CPM-based optimization algorithm, the idea of the algorithm is that after finding all the (k-clique) subgraphs in the network, these subgraphs are transformed into meta-nodes, and subsequently, those nodes that are not delineated are moved, and the attribution of these nodes is determined by analyzing the change in the modularity degree resulting from the movement of the nodes. In general, for the CPM algorithm and its optimization algorithms, the choice of the k-value has a more significant impact on the community delineation results, especially when the k-value is lower in some sparse networks, which tends to create an excessive number of small communities.

**Based on label propagation methods**. CORPA [21] is the first algorithm to use the LPA idea in overlapping community detection, which makes the nodes retain multiple labels during label propagation, and then determines the division of overlapping communities based on these labels, but the algorithm randomly selects nodes for label propagation, which leads to the problem of low accuracy of community division as well as the unstable division results; WLPA [22] avoids the problem of label oscillation caused by synchronization in the label propagation phase of the algorithm by means of “asynchronous + ascending”, and also takes into account the difference in the influence of different nodes in the network, but only refers to the node degree when considering the influence of the nodes, which is unreasonable and cannot effectively reflect the node’s influence. NALPA [23] assigns four types of capabilities to the nodes in the network, and influences the importance of the nodes in the network as well as constructs a new label propagation mechanism using the four abilities, but its high time complexity makes it difficult to deal with large-scale networks efficiently; FLPA [24] adopts graph compression technology to merge sparse nodes in the network, which reduces the network size, and then optimizes the label propagation process based on node influence and label weight to improve the accuracy of community detection, but it is ineffective in dealing with dense networks because it involves the image compression strategy, which limits the application of the algorithm in specific network. INF-CORPA [25] integrates node “degree” and “k-core” theories, assigns influence to nodes, sets thresholds, and controls label propagation to reduce erroneous labels, but the label thresholds may be set differently in different networks, and the ability of the algorithm to deal with label propagation interference needs to be enhanced for some complex community structure.

**Based on local expansion and optimization methods**. OSLPM [26] is the first algorithm that can simultaneously handle directed bandwidth weighted networks and be able to identify overlapping communities, which expands the subpopulations by means of a kind of local optimization of fitness function and obtains the community structure from the expansion; Whang et al. [27] identifies the seed set by Graclus centers, and then based on the optimized PageRank strategy personalized expansion to detect overlapping communities in the network; Ding X et al [28] overcame the problem of poor fault tolerance by optimizing node affiliation to discard low-quality seeds and communities, but had limited effect in identifying highly aggregated communities and high computational cost in large-scale networks.

**Based on link partition-based methods**. For this solution, the network is considered as a kind of link graph that can be processed by a clustering algorithm, by processing the links in the network instead of the nodes, and then community detection is performed based on this link graph [29]. Ahn et al. [14] mapped the initial network structure onto a link graph, and then iteratively merged the similar links, and then determined the optimal partition hierarchy, and finally restored the edge graph as a node-with-node clusters. In summary Link Partition based Method can easily detect overlapping nodes, but its reliability has not been properly proven due to its fuzzy definition of community- based [30].

**Based on machine learning methods**. Berahmand K et al. [31] proposed a semi-supervised deep attribute clustering framework based on dual autoencoders. By integrating structural and attribute information into a dual-view representation and combining it with pairwise constraint optimization, the framework significantly improves clustering performance in attributed networks. DSSC [32] effectively enhances the accuracy and efficiency of community detection in complex networks through the design of a semi-autoencoder and a pairwise constraint matrix. Zhou et al. [33] proposed CDBNE, an unsupervised attributed network embedding framework for community detection. It integrates graph-attentive autoencoders to model network topology and node attributes, combines encoder/decoder outputs with mesoscopic community priors, and employs self-training clustering to optimize node representations. CPGC [34] improves graph convolution operations and incorporates community-oriented similarity by integrating representation learning with clustering. It effectively utilizes both attribute and structural information to detect overlapping and non-overlapping communities. However, there is still room for improvement in terms of computational efficiency and scalability. GEAM [35] is a graph-enhanced attention model designed for multilayer networks. It effectively integrates cross-layer semantic information and improves community detection accuracy through inter-layer contrastive learning, an adaptive fusion mechanism, and an edge density-driven module.

In addition, there are many other various overlapping community detection methods such as fuzzy-based detection methods [36], agent-based methods [37], and modularity optimization-based methods [38]. These methods implement overlapping community detection from various perspectives of the network.

## Solution for OSFCM

Fuzzy cluster analysis refers to solving clustering problems using fuzzy mathematical methods. It quantifies uncertainty in sample-category assignments, captures transitional memberships, and reflects real-world complexity more objectively, thus it has become the mainstream of the research of cluster analysis.

Typical steps of the cluster analysis based on fuzzy relationships: data specifications, construction of fuzzy similarity matrices and fuzzy classification. Among the various algorithms for fuzzy classification, the one with higher real-time requirements is the fuzzy clustering method based on the objective function, which reduces the clustering to a nonlinear planning problem with constraints, and obtains the fuzzy division and clustering of the dataset by optimizing the solution. An optimization scheme for selecting generations to minimize this objective function is the well-known hard C-means(HCM) algorithm. An organizational data analysis technique derived from the optimization of the hard clustering objective function is the fuzzy C-means, which can be transformed into an optimization problem and solved with the help of nonlinear programming theory of classical mathematics.

**Fig 1:**
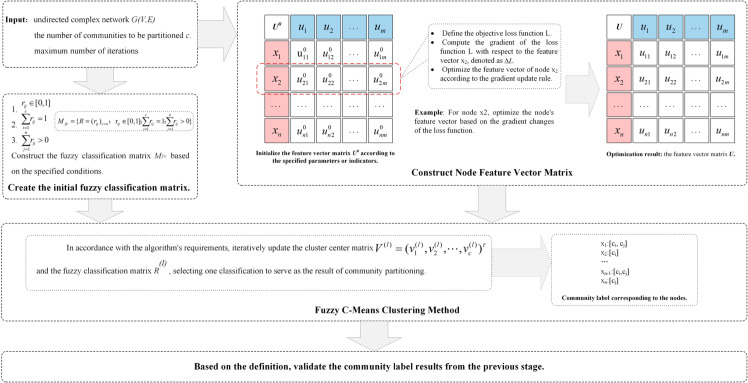
The process of OSFCM.

Among the clustering algorithms based on objective function, fuzzy c-means (FCM, fuzzy c-means), also known as ISODATA algorithm, i.e., iterative self-organizing data analysis technique, is constructed to solve the clustering problem with the help of the objective function method, which uses the mean-squared approximation theory to construct a constrained nonlinear programming function. In the following we discuss the core theory of the algorithm developed in the paper, the process of OSFCM is showed in Fig 1.

### Matrix construction for fuzzy classification

Given that the set of nodes of the network is $X=\{x_{1},x_{2},\ldots ,x_{n}\}$, where each node *x*_*i*_ has *m* characteristic indicators, $u_{i}=\{u_{i1},u_{i2},\ldots ,u_{im}\}$, if *X* is to be classified into *c* classes, each of its classification results corresponds to a Boolean matrix of order $R=(r_{ij}{)}_{c\times n}$, where *n* is the number of nodes, and *r*_*ij*_ is as follows:

$$r_{ij}=\left\{ \begin{array}{c} 1,\;x_{i}\text{ belongs to the }C_{i}\;,\\ 0,\;\text{otherwise.} \end{array}\right.$$ (1)

For example, given that $X=\{x_{1},x_{2},\ldots ,x_{n}\}$ denotes the set of classified nodes, if the classification result is $\left\{x_{1},x_{3}\right\}$, $\left\{x_{2},x_{5}\right\}$, {*x*_4_}, then the corresponding classification matrix is:

$$R=\left[\begin{array}{ccccc} 1 & 0 & 1 & 0 & 0\\ 0 & 1 & 0 & 0 & 1\\ 0 & 0 & 0 & 1 & 0 \end{array}\right]$$ (2)

$R=(r_{ij}{)}_{c\times n}$ satisfies the following conditions: (1) $r_{ij}\in \{0,1\}$; (2) $\sum_{i=1}^{c}r_{ij}=1$, that is, each column has and only one element of 1 and the rest of the elements are 0, ensuring that each object can and must belong to only one of the categories; (3) $\sum_{j=1}^{n}r_{ij}>0$, that is, the sum of the elements of each row is greater than 0, ensuring that each category is not empty and that there can be more than one object in a category. Any matrix *R* satisfying the above-mentioned 3 properties corresponds to a classification.

Given that *M*_*c*_ is the whole c×n matrix *R* satisfying the above condition, then *M*_*c*_ contains all the possible classification results of *X* being classified into class *c*. So we call *M*_*c*_ the classification space of the node set *X* being classified into class *c*, which is defined as:

$$M_{c}=\{R=(r_{ij}{)}_{c\times n}:r_{ij}\in \{0,1\};\;\sum_{i=1}^{c}r_{ij}=1;\;\sum_{i=1}^{n}r_{ij}>0\}$$ (3)

Based on the above-mentioned matrices for classification, we construct fuzzy classification matrices. A fuzzy classification takes the objects *x*_*i*_ in the classified *X* as belonging to a certain class with different degrees of affiliation, so each class is considered as a fuzzy subset on *X*. Thus each fuzzy classification is a fuzzy matrix $R=(r_{ij}{)}_{c\times n}$ of size c×n, which satisfies the following conditions:

1. $r_{ij}\in [0,1]$, that is, the classification matrix elements take values between 0 and 1, not 0 or 1, indicating that a node can simultaneously belong to multiple communities to varying degrees;
2. $\sum_{i=1}^{c}r_{ij}=1$, for each individual node, the sum of its membership degrees across all communities must equal 1;
3. $\sum_{i=1}^{n}r_{ij}>0$, that is, all nodes must exhibit non-zero membership in at least one community, ensuring no community is entirely unpopulated.

Any fuzzy matrix *R* that meets the above conditions corresponds to a fuzzy categorization of *X* into *c* classes.

Denote *M*_*c*_ as the whole of the fuzzy matrix *R* satisfying the above three properties, which is called the fuzzy classification space where *X* is classified into *c* classes, then is defined as:

$$M_{c}=\{R=(r_{ij}{)}_{c\times n}:r_{ij}\in [0,1];\;\sum_{i=1}^{c}r_{ij}=1;\;\sum_{i=1}^{n}r_{ij}>0\}$$ (4)

### Construct node feature vector matrix

Given that the feature vector matrix consisting of the characteristic metrics of all the nodes in the network is denoted as $U=(u_{ij}{)}_{n\times m}$. For the fuzzy C-means clustering method, the characteristic indexes of the nodes not only determine how the nodes are finally grouped, but also influence each step of the clustering process. Thus it is especially important to choose appropriate characteristic indexes to construct the feature vector matrix. In OSFCM, we select three widely used feature metrics in network analysis: Degree, Degree Centrality, and Betweenness Centrality. These metrics effectively reflect a node’s position and relative importance within the network structure. Their definitions are as follows:

degree centrality:

$$DC(u)=\frac{d_{u}}{n-1}$$ (5)

betweenness centrality of nodes:

$$BC(u)=\frac{2}{\left(n-1)(n-2\right)}\sum_{s\neq u\neq t}\frac{n_{st}^{u}}{g_{st}}$$ (6)

where $n_{st}^{u}$ denotes the number of paths passing through node *u* connecting *s* and *t*, and which are shortest paths, and *g*_*st*_ denotes the number of shortest paths connecting *s* and *t*.

However, directly using these metrics as clustering inputs may result in communities that overly rely on node importance. For example, all nodes with high centrality may be grouped into the same community, ignoring the potential lack of actual connections between them. Therefore, to improve the rationality of community division, we introduce an optimization objective function based on a node similarity matrix. Specifically, we aim to design an objective loss function L and a node similarity matrix S, to achieve that for similar nodes (denoted by larger *S*[*u*,*v*]), the difference ||U[u]−U[v]|| of their eigenvectors should be as small as possible, and for dissimilar nodes (denoted by smaller *S*[*u*,*v*]), the difference ||U[u]−U[v]|| of their eigenvectors should be as large as possible, and the objective loss function *L* is specifically defined as:

$$L=\sum_{u,v}S[u,v]\cdot ||U[u]-U[v]|{|}^{2}$$ (7)

The objective is to minimize *L*, which ensures that nodes with high similarity remain close in the feature space while weakening the connections between nodes with low similarity. The node similarity matrix is defined as:

$$S(u,v)=\frac{|N_{u}\cap N_{v}|+1}{N_{u}}$$ (8)

Where *N*_*u*_ denotes the set of neighboring nodes of node *u* and *S*(*u*,*v*) reflects the influence of exerted on u by its neighbors. In the formula, the numerator is incremented by 1 because the intersection of the neighboring node sets of nodes u and v does not include node u itself. In real-world social networks, when nodes u and v share no other common neighbors, the value of $\left|N_{u}\;\cap \;N_{v}\right|$ is 0. However, node u itself can be considered a common neighbor of the two.

To optimize the target loss function, we use the gradient descent method, the gradient of the loss function L with respect to the feature vector U [u] is defined as:

$$\Delta L(u)=2\sum_{u,v}S(u,v)\cdot (U[u]-U[v])$$ (9)

Based on this, the feature vector update formula is defined as:

$$U[u{]}_{new}=U[u{]}_{old}-\eta \cdot \Delta L(u)$$ (10)

Where *η* denotes the learning rate with a value of 0.001. When $||U[u{]}_{new}\;-\;U[u{]}_{old}||\leq 0.01$ or the maximum number of iterations *t* is reached, it indicates that the update of the feature vector has achieved an acceptable result. By performing gradient optimization on the initial feature vector matrix, the node characteristics are optimized so that these characteristics can, to some extent, reflect community information of the nodes, thereby avoiding the inclusion of unrelated nodes in the same community. The construction process of the feature vector matrix *U* is given in Algorithm 1.

**Algorithm 1: Construct node feature vector matrix.**

**Input**: degree centrality:DC

betweenness centrality of nodes:BC

**Output**: Node Feature Vector Matrix: U

1: $U_{init}\leftarrow DC,BC$

2: **for** *each node u in V* **do**

3:    **for** each node v in V **do**

4:     S(u,v) ← similarity index of node *u* to v according

to Eq 8

5:    **end for**

6: **end for**

7: **repeat:**

8:    **for** each node u in V **do**

9:     Update *U*[*u*] according to Eq 10

10:    **end for**

11: **until:**

12:    **For any u**, $||U[u{]}_{new}-U[u{]}_{old}||\leq 0.01$

13:    **or** $t=t_{max}$

14: **return U**

### Fuzzy C-means clustering method

Divide the node set *V* into *c* classes, and let the *c* cluster center vectors form the matrix *V*.

$$V=\left[\begin{array}{c} v_{1}\\ v_{2}\\ \vdots \\ v_{c} \end{array}\right]=\left[\begin{array}{cccc} v_{11} & v_{12} & \ldots & v_{1m}\\ v_{21} & v_{22} & \ldots & v_{2m}\\ \vdots & \vdots & & \vdots \\ v_{c1} & v_{c2} & \ldots & v_{cm} \end{array}\right]$$ (11)

In order to obtain an optimal fuzzy classification, the following clustering criterion is used to select the best fuzzy classification from the fuzzy classification space *M*_*fc*_. Find the appropriate fuzzy classification matrix *R* and cluster center matrix *V*, so that the following objective function *J*(*R*,*V*) reaches the minimum value.

$$J(R,V)=\sum_{k=1}^{n}\sum_{i=1}^{c}(r_{ik}{)}^{q}||u_{k}-v_{i}|{|}^{2}$$ (12)

Where fuzzy parameter*q* = 2, $u_{k}\;-\;v_{i}$ denotes the distance between the feature vector *u*_*k*_ of an object *x*_*i*_ and the clustering center vector of class *i*. In addition, we expect the classes to be tightly clustered and not too spread out, and we expect the objects in each class to have the smallest sum of the squared distances to the corresponding cluster center. Obviously, *J*(*R*,*V*) is related to the setting of these *c* clustering centers $\left\{v_{1},v_{2},\ldots ,v_{c}\right\}$, and the smaller the distance is, the better the clustering effect is.

Generally speaking, solving the objective function *J*(*R*,*V*) is challenging, and its approximate solution is usually obtained by iterative operations. When $1\leq q,u_{k}\neq v_{i}$ is given, we use the fuzzy mean algorithm to perform the iteration, and the process is convergent, the specific steps are as follows:

(1) Select the number of classifications *c*, 2≤c≤n, take an initial fuzzy classification matrix $R^{\left(0\right)}\in M_{fc}$, iterate step by step l=0,1,2,…. For ${\text{R}}^{\left(\text{l}\right)}$, compute the cluster center matrix $V^{\left(l\right)}={\left(v_{1}^{\left(l\right)},v_{2}^{\left(l\right)},\ldots ,v_{c}^{\left(l\right)}\right)}^{T}$, and correct the fuzzy classification matrix ${\text{R}}^{\left(\text{l}\right)}$, where:

$$v_{i}^{\left(l+1\right)}=\frac{\sum_{k=1}^{n}(r_{ik}^{\left(l\right)}{)}^{q}\cdot u_{k}}{\sum_{k=1}^{n}(r_{ik}^{\left(l\right)}{)}^{q}}$$ (13)

$$r_{ik}^{\left(l+1\right)}=[\sum_{j=1}^{c}(\frac{\left||u_{k}-v_{i}^{\left(l\right)}|\right|}{\left||u_{k}-v_{j}^{\left(l\right)}|\right|}{)}^{\frac{2}{\left(q-1\right)}}{]}^{-1}$$ (14)

(2) Compare ${\text{R}}^{\left(\text{l}\right)}$ with *R*^(*l* + 1)^. If $max\{|r_{ik}^{\left(l+1\right)}\;-\;r_{ik}^{\left(l\right)}|\}\leq \varepsilon$, for a given precision ε=0.001, then $R^{\left(l\;+\;1\right)}$ and ${\text{v}}^{\left(\text{l}\right)}$ are the desired results and the iteration stops, otherwise, l=l+1 and return to step 1, repeat the process. The solving process is given in Algorithm 2.

**Algorithm 2: Fuzzy C-means clustering method.**

**Input**: X, c, q, 𝜖

**Output**: the fuzzy classification matrix $R_{c\times n}$

the cluster center matrix $V_{c\times m}$

1: $R^{0}\leftarrow M_{fc}$

2: **repeat:**

3:   **for** i = 1 to c **do**

4:    Update $v_{i}^{\left(l+1\right)}$ according to Eq 13

5:   **end for**

6:   **for** k=1 to n **do**

7:    **for** i = 1 to c **do**

8:     Update $r_{ik}^{\left(l+1\right)}$ according to Eq 14

9:    **end for**

10:   **end for**

11:   **if** $max\{|r_{ik}^{\left(l+1\right)}-r_{ik}^{\left(l\right)}|\}\leq \epsilon$ **then**

12:    **return** $V_{c\times m}$, $R_{c\times n}$

13: **until: t** = *t*_*max*_

14: **return** $V_{c\times m},R_{c\times n}$

The fuzzy classification matrix and the cluster center matrix obtained by applying the above algorithm are locally optimal solutions with respect to the number of classifications, the initial fuzzy classification matrix, and the parameter q.

(3) After obtaining the optimal fuzzy classification matrix and the optimal cluster center matrix that meet the requirements, the classification is carried out according to the following discriminative principles:

1. Using the optimal fuzzy classification matrix clustering, given the optimal fuzzy classification matrix is $R^{*}=(r_{ik}^{*}{)}_{c\times n}$, for any *x*_*k*_, in the k-th column of ${\text{R}}^{*}$, if $R^{*}\geq \tau \cdot max\{r_{ik}^{*},1\leq j\leq c\}$, then the object will be attributed to the i-th class, i.e., the object *x*_*k*_ is assigned to the class with the higher membership degree.
2. Using the optimal cluster center matrix for clustering: let the obtained best clustering center matrix be $V^{*}={\left(v_{1}^{*},v_{2}^{*},\ldots ,v_{c}^{*}\right)}^{T}$. For any *x*_*k*_, if its corresponding feature vector *u*_*k*_ meets $\left||u_{k}-v_{i}^{*}||\leq (1-\tau )\cdot min\{||u_{k}-v_{j}^{*}||,1\leq j\leq c\right\}$, then the object *x*_*k*_ is assigned to the class *i*.

Since this algorithm requires $u_{k}\neq v_{i}$, the selection of the initial fuzzy classification matrix ${\text{R}}^{\left(0\right)}$ must comply with the three conditions of the fuzzy classification matrix, but also has the following restrictions: (a) The initial matrix ${\text{R}}^{\left(0\right)}$ cannot be a constant matrix where all elements are the same; (b) The initial matrix ${\text{R}}^{\left(0\right)}$ cannot be a matrix with the same value of the elements of a row. For class with only one object in the initial matrix, it should be removed before clustering and reinserted after clustering.

### Label verification

After the initial community division by FCM, the node feature vector matrix may not fully capture community structure due to its dependence on node-level metrics. Thus, residual errors persist in the detection results. To further improve the accuracy of community detection, this paper introduces a calibration mechanism based on community attraction force, which leverages the influence of neighboring nodes to refine community labels. Specifically, if multiple neighboring nodes of a given node u belong to a particular community ci and these neighbors exert strong influence on u, then community ci is considered to have a significant “attraction" effect on node u. We define the attraction force between node u and community *c*_*i*_ as:

$$F(u,c_{i})=\sum_{v\in N_{u},v\in c_{i}}S(u,v)$$ (15)

Where *F*(*u*, *c*_*i*_) denotes the traction of community *c*_*i*_ on node *u*.

The greater the attraction F(u,*c*_*i*_), the stronger the “pull” exerted by the neighbors in community *c*_*i*_ on node u.

To determine whether node u belongs to a candidate community *c*_*i*_, we set an attraction threshold. Let *F*_*max*_(*u*) be the maximum attraction among all candidate communities of node u. We use $0.9\times F_{max}(u)$ as the threshold for decision-making. When $0.9\times F_{max}(u)\leq F(u,c_{i})$, node u is considered to belong simultaneously to community *c*_*i*_.

This strategy uses a unified relative threshold to determine node membership in different communities, avoiding over- or under-assignment in community detection. Although the threshold is heuristically set, it has demonstrated good adaptability and stability across multiple datasets. In future work, we plan to introduce an adaptive attraction criterion to make the calibration process more theoretically grounded and flexible.

In summary, the specific workflow of the OSFCM algorithm is shown in Algorithm 3.

**Algorithm 3: OSFCM Algorithm.**

**Input**: $G(V,E),c,t_{max},\eta ,q,\epsilon$

**Output**: Community set C

1: construct matrix for fuzzy classification *M*_*fc*_

2: U←invoke Algorithm 1

3: $V_{c\times m},R_{c\times n}\leftarrow \text{invoke Algorithm 2}$

4: **for** each node u in V **do**

5:    Classify node $u\;\text{based}\;\text{on}\;V_{c\times m}\;\text{or}\;R_{c\times n}$

6: **end for**

7: **repeat:**

8:    **for** each node u in V **do**

9:     Validate node labels according to Eq 15

10:    **end for**

11: **until:** community set C no longer changes.

12: **return C**

### Complexity analysis

Given an undirected unweighted graph G, where |V| is the number of nodes, |E| is the total number of edges, c is the number of communities, and t is the number of iterations, the time complexity of each step in the OSFCM algorithm is calculated as follows:

Step 1: Create the initial fuzzy partition matrix and time complexity is O(c×|V|);

Step 2: Construct the similarity adjacency matrix and time complexity is $O(|V{|}^{2})$;

Step 3: Construct the node feature vector matrix and time complexity is $O(t\times |V{|}^{2})$;

Step 4: Apply the fuzzy c-means clustering method and time complexity is $O(t\times |V{|}^{2})$;

Step 5: Perform label verification and time complexity is $O(|V{|}^{2})$.

Therefore, we get the time complexity of OSFCM algorithm which is

$O(c\times |V|)+O(|V{|}^{2})+O(t\times |V{|}^{2})+O(t\times |V{|}^{2})+O(t\times |V{|}^{2})\approx O(t\times |V{|}^{2})$

## Experimental results and analysis

### Test sets and comparison algorithms

To validate the performance of OSFCM for overlapping community detection, it is validated and analyzed with six other overlapping community detection algorithms (COPRA [21], FLPA [24], CPM [18], LINK [29], AOCD [39], CDMG [40]) on several network datasets. In addition, all algorithms were implemented using python and executed on a processor Intel(R) Core(TM) i5- 13400 CPU@2.5 GHz with 32.00 GB of RAM in Windows 10 environment. In order to eliminate randomness, all experiments were repeated 100 times and averaged under the same conditions.

We selected seven in many real network datasets, including Zachary Karate Club dataset [41], dolphin dataset [42], polbooks dataset, football dataset [3], Jazz dataset [43], Email dataset and PGP dataset [44], these datasets node sizes cover both small-scale and large-scale networks. The number of nodes (Nodes), node relationships (edges) of these network datasets are given in Table 1. Where ‘-\-’ indicates that there is no real network community division on this dataset.

**Table 1:** Details of Real-World Network Datasets.

**Networks**	Karate	Dolphin	Polbooks	Football	Jazz	Email	PGP
**Nodes**	34	63	105	115	198	1133	10681
**Edges**	78	159	441	613	2742	5451	24316
*C*_*n*_	2	3	3	12	-\-	-\-	-

For synthetic networks, we selected the LFR benchmark [45], which is widely used in the field of community detection with various adjustable parameters, including node count, node degree distribution (average node degree $k_{ave}$, maximum node degree *k*_*max*_), and community size (minimum community size *C*_*min*_ and maximum community size *C*_*max*_), power distribution index of node degree and power distribution index of community size(τ1,τ2).

In addition, the ambiguity of community boundaries can be affected in LFR networks by adjusting the mixing parameter *μ*. A higher *μ* value increases community ambiguity, making detection more challenging. We conduct experiments on four different sizes of LFR networks (LFR1, LFR2, LFR3, LFR4) to verify the effectiveness of the algorithm in OSFCM. The detailed parameter settings of specific LFR networks are shown in Table 2.

**Table 2:** Parameter Settings for LFR Networks.

Parameter	Node	$k_{ave}$	*k*_*max*_	*C*_*min*_	*C*_*max*_	τ1	τ2
**LFR1**	1000	20	50	10	50	2	1
**LFR2**	5000	20	50	20	100	2	1
**LFR3**	10000	20	50	20	100	2	1
**LFR4**	20000	30	80	20	100	2	1

### Evaluation metrics

Modularity (Q) is a metric defined from the global perspective of the network to quantify the topological characteristics of communities, which is often used to evaluate the merits of the community segmentation results, and is applicable to non-overlapping communities. For the evaluation of overlapping community detection results, the improved modularity evaluation metric EQ is used, and the value range is generally [0,1], the higher the value of EQ indicates that the network segmentation results are better, with the closer connection within the community and the sparser connection between the communities. Specifically defined as:

$$EQ=\frac{1}{2m}\sum_{i}^{c}\sum_{u\in C_{i},v\in C_{i}}\frac{1}{O_{u}O_{v}}[A_{uv}-\frac{d_{u}\cdot d_{v}}{2m}]$$ (16)

Where *m* denotes the number of edges between all the nodes in the network *G*, *C* denotes the number of communities, *u* and *v* denote two nodes in the community *C*_*i*_, *O*_*u*_ denotes the number of communities belonging to the node *u* (number of groups), *A* denotes the adjacency information matrix, $A_{uv}=1$ when there is an edge connecting nodes *u* and *v*, otherwise $A_{uv}=0$. *d*_*u*_ denotes the degree of the node *u*.

Normalized Mutual Information (NMI) is an evaluation index that can effectively assess the accuracy of the community discovery algorithm by comparing it with the real true value of the social network, which takes the value in the range of [0,1], and the higher the value indicates that the more accurate the community division results are, and when NMI=1, it indicates that the division result is exactly the same as the real true value, which is specifically defined as:

$$NMI(A,B)=\frac{2\sum_{i=1}^{C_{A}}\sum_{j=1}^{C_{B}}N_{ij}log(\frac{N_{i.}N_{.j}}{N_{ij}N})}{\sum_{i=1}^{C_{A}}N_{i.}log(\frac{N_{i.}}{N})+\sum_{j=1}^{C_{B}}N_{.j}log(\frac{N_{.j}}{N})}$$ (17)

where *A* denotes the actual community segmentation result, *B* denotes the experimentally measured segmentation result, *C*_*A*_ denotes the number of communities of *A*, and *C*_*B*_ denotes the number of communities of *B*, *N* denotes the segmentation information matrix, the rows in the matrix denote the actual communities, and columns denote the detected communities, *N*_*ij*_ denotes the number of vertices that appear in the community *i* and are also contained in the detected community *j*, *N*_*i*._ denotes the sum of the i-th row of the segmentation information matrix, and *N*_.*j*_ is summed up over the jth column.

### Experimental parameter analysis

**Fig 2:**
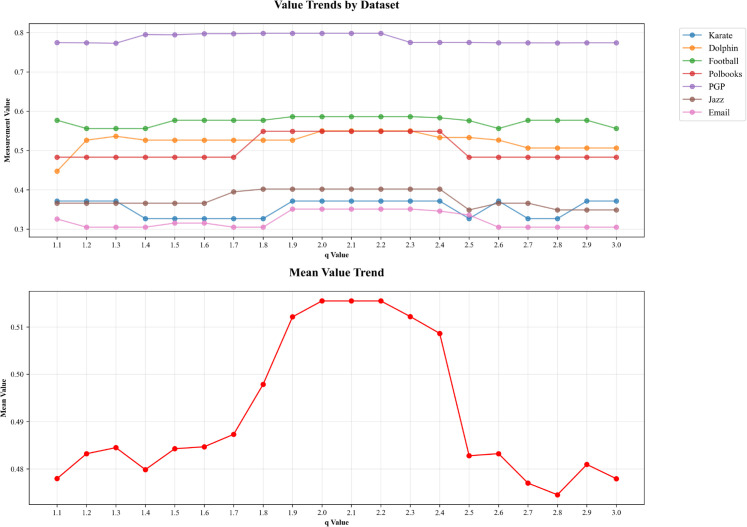
Analysis and comparison of q parameter results.

The fuzzy parameter q controls the degree of fuzziness in the membership assignments during the clustering process: smaller values of q produce crisper clustering results, while larger values allow for greater fuzziness. To evaluate the sensitivity of the proposed method to the fuzzy parameter q, we conducted a series of experiments on datasets such as Karate Club and Football. In these experiments, all other parameters were kept constant, and only the value of q was varied within the range from 1.1 to 3. The experimental results are shown in the Fig 2.

As shown in Fig 2, when the fuzzy parameter q varies within the range of [1.1, 1.5], the performance of the algorithm shows an upward trend across all datasets, without exhibiting relatively stable behavior. In contrast, within the range of [1.9, 2.3], the algorithm demonstrates strong parameter robustness and achieves its best performance on all datasets. However, when q becomes too large, the performance slightly declines. This is because a low q value leads to overly rigid community partitions, while an excessively high q causes the membership degrees of nodes to become too vague, thereby affecting detection accuracy. In summary, the algorithm shows good adaptability in practical applications when q is set around 2.1. In this study, we set q = 2.0.

### Analysis of experimental results

#### Analysis of real-world network detection results.

In order to verify the effectiveness of OSFCM in practical applications, we compare OSFCM with several other comparative algorithms on 8 real network datasets. The experimental results are shown in Table 3, which gives the corresponding EQ values of overlapping community modularity.

**Table 3:** Extended Modularity (EQ) Values for Overlapping Communities.

Datasets	CORPA	FLPA	CPM	LINK	AOCD	CDMG	OSFCM
KarateClub	0.3039	**0.3715**	0.1321	0.2481	0.3659	0.3582	**0.3715**
Dolphin	0.2832	0.5068	0.2054	0.2353	0.5487	**0.5581**	0.5501
Polbooks	0.2981	0.4980	0.3152	0.2279	0.5395	0.5082	**0.45491**
Football	0.3692	0.5631	0.1511	0.2602	0.5720	0.5482	**0.5863**
Jazz	0.2021	0.3983	0.1575	0.1462	0.3782	0.3681	**0.4021**
Email	0.1641	0.3421	0.2902	0.1951	0.3291	0.3159	**0.3512**
PGP	0.5588	**0.802**	0.5478	0.3864	0.5308	0.5106	0.7982

As can be seen from Table 3, the detection results of the algorithm proposed in OSFCM are better than FLPA, CORPA, CPM and LINK algorithms on all these datasets. For the karate dataset, although the average modularity value of the CPRPA algorithm is lower than that of OSFCM and the FLPA algorithm, the CORPA algorithm can achieve a value of about 0.382 in individual cases during the actual validation process. However, it is shown based on the actual test that when the modularity of the karate dataset is 0.3715, the community division results obtained from the experiment are consistent with the actual community division results (although there are no overlapping community nodes in the actual division), which indicates that OSFCM does not divide the karate community into overlapping communities but achieves the community detection results consistent with the actual division, which verifies that OSFCM is effective in actual application. In addition, we can see from the table that among all the tested algorithms, FLPA algorithm achieves higher EQ value on PGP network, and the CDMG algorithm performs well on the Dolphin network, but the EQ value of OSFCM is not much different from theirs, and the detection results of OSFCM are much better than FLPA algorithm on other test sets. For the FLPA, CORPA, LINK, and AOCD algorithms, their detection results are inferior to those of the proposed algorithm across all datasets. The results in the table show that OSFCM can obtain a better community structure both on small-scale networks and large-scale networks, which verifies the effectiveness of OSFCM.

#### Analysis of LFR network detection results.

For the LFR network, the initialization of the LFR network is accompanied by the initialization of the community information contained in the nodes, providing not only the LFR network dataset, but also the real LFR network segmentation results. At this point, the NMI evaluation metrics are used to validate the effectiveness of OSFCM in terms of the accuracy of community segmentation.

**Fig 3:**
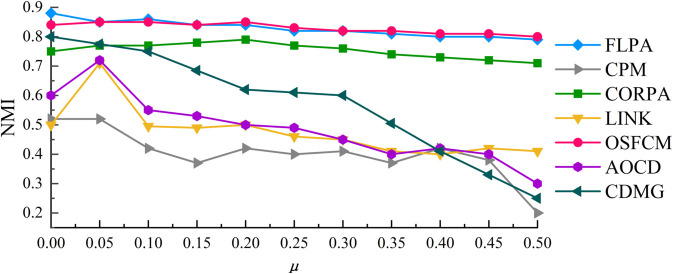
Community detection results on the LFR1 network dataset.

The experimental results of OSFCM with other comparative algorithms on the LFR1 artificial network dataset are shown in Fig 3. It shows the variation curve of NMI with *μ*, where *μ* increases from 0.1 to 0.5 in increments of 0.05 to ensure the accuracy of the test results.

From Fig 3, it can be seen that FLPA has the highest NMI value when the value of *μ* is 0.0, but in the interval after that, both OSFCM and FLPA can obtain better community detection results, with the NMI values centered around 0.85, and the results of OSFCM are even better than the other algorithms on the interval [0.3,0.5]. For CORPA algorithm, its NMI value can also be maintained around 0.75 on the whole interval, and better community detection results can also be obtained. The CDMG algorithm can achieve an NMI value of around 0.8 when μ=0.0, but as the *μ* value increases, the NMI value drops rapidly. However, for CPM algorithm and LINK algorithm, their community detection results are poorer and the interval fluctuates more, and most of the NMI values of these two algorithms are less than 0.5. From the figure, it is intuitively observed that the OSFCM is significantly better than the other algorithms although the gap between the detection results and the FLPA algorithm is not large, which indicates that our algorithm can effectively detect meaningful overlapping community structures.

**Fig 4:**
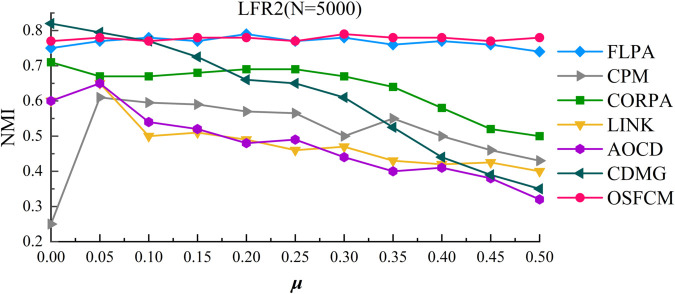
Community detection results on the LFR2 network dataset.

The experimental results of OSFCM and other comparative algorithms on the LFR2 artificial network dataset are shown in Fig 4. The curves of NMI versus *μ* are demonstrated, where *μ* is increased from 0.1 to 0.5 in increments of 0.05 to ensure the accuracy of the test results.

From Fig 4, it can be seen that when the value of *μ* is in the interval [0,0.25], the NMI values of OSFCM and FLPA are approximate, and both of them are maintained around 0.77, which can obtain better community detection results, but in the interval after that, the detection results of OSFCM are better than those of the FLPA algorithm, and the two algorithms are significantly better than the other algorithms on the whole interval. The CDMG algorithm achieves higher NMI than the FLPA and OSFCM algorithms when *μ* is small, but its NMI becomes lower than both when μ>0.1. For CORPA algorithm, when the value of *μ* is less than 0.3, the value of NMI can reach 0.7, but with the increase of u, its detection results also begin to drop significantly, and it is difficult to maintain at about 0.7, while LINK algorithm and AOCD algorithm can reach more than 0.6 only at the beginning, and the value of NMI begins to plummet with the increase of u, resulting in poor detection results. Moreover, the LINK algorithm and CPM algorithm are greatly affected by the value of u, and the NMI value of these two fluctuates greatly with the change of u. Therefore, by comparison and analysis, OSFCM is less affected by the value of *μ* and can achieve better community detection results, and the algorithm is more applicable, which verifies the validity of the algorithm calculated in OSFCM.

**Fig 5:**
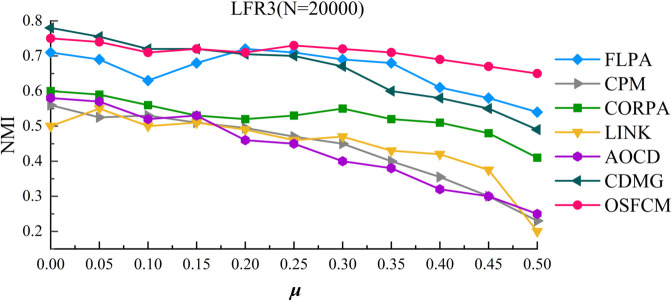
Community detection results on the LFR3 network dataset.

The experimental results of OSFCM and other comparative algorithms on the LFR3 artificial network dataset are shown in Fig 5,demonstrating the curve of NMI with *μ*, where *μ* increases from 0.1 to 0.5 in increments of 0.05.

As can be seen from the figure, the FLPA algorithm begins to show larger fluctuations in the value of NMI compared to the previous dataset, and the results of NMI as a whole are poorer compared to OSFCM. On the whole interval, FLPA can obtain detection of little difference with OSFCM only when *μ* obtains individual values, while in other cases, OSFCM is significantly better than the FLPA algorithm. Similar to previous observations, the CDMG algorithm achieves a higher NMI than the OSFCM algorithm when *μ* is small, but its NMI becomes significantly lower than that of OSFCM when μ>0.15. As for CPM, CORPA, and LINK algorithms, the detection results of the three algorithms are similar, and all of them achieve an NMI value of about 0.55 initially, while the NMI values of these algorithms decrease with the increase of u. Even the NMI values of CPM and LINK algorithms reach 0.2 when *μ* equals to 0.5. Obviously, the detection results of these algorithms gradually deteriorate as the size of network increases, and the accuracy of community segmentation also begins to gradually decline. In comparison, the stability of the detection results of OSFCM is better than other algorithms in both large-scale and small-scale networks, which verifies the effectiveness of OSFCM.

**Fig 6:**
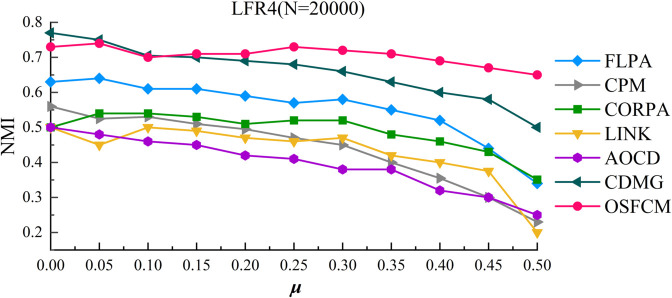
Community detection results on the LFR4 network dataset.

The experimental results of OSFCM and other comparative algorithms on the LFR4 artificial network dataset are shown in Fig 6. demonstrating the curve of NMI with u, where *μ* increases from 0.1 to 0.5 in increments of 0.05.

As can be seen from the figure, compared with the LFR1, LFR2, and LFR3 datasets, on the LFR4 dataset, the community detection results of the FLPA algorithm are not as good as before, while OSFCM can still maintain better community detection results, although the value of the NMI only reaches about 0.7, the results are still a significant advantage for other algorithms. Moreover, the results achieved by OSFCM are relatively stable throughout the test interval, while all other algorithms decrease with the increase of *μ* value. Even when *μ* is equal to 0.5, the NMI values of the other four algorithms are less than 0.4, which indicates that most community detection algorithms are difficult to achieve better community delineation results as the community results are more ambiguous and the size of community nodes is larger. Therefore, our algorithm can effectively detect meaningful overlapping community structures even in large-scale, more ambiguous community structures compared to other algorithms, which verifies the effectiveness of OSFCM.

## Conclusion

Aiming at the complex and diverse community detection tasks, this paper proposes an overlapping community optimization method (OSFCM) based on network structural characteristics and FCM. Firstly, the structural characteristics of the nodes in the network are abstracted into the characteristic indexes of the nodes and the feature vector matrix is constructed, then, the feature vector matrix is optimized based on the proposed gradient optimization method of the objective function, secondly, the preliminary community delineation results are generated based on the optimized feature vector matrix and fuzzy C-means clustering analysis method, and finally, the community membership of each node is verified. Comparing and analyzing OSFCM with several other overlapping community detection algorithms, simulation results on real and synthetic network datasets show that OSFCM algorithm exhibits good modularity advantage on the given dataset and has higher NMI accuracy in both small and medium-large synthetic network.

In the future, we will continue to explore application scenarios for large-scale community detection algorithms and apply our solution to dynamic or weighted networks to maximize its advantages in solving large-scale complex problems.

## Supporting information

S1 File Network structure dataset. (CSV)

S2 File Community label of the node. (CSV)

S3 File Experimental division results of the Karate network. (CSV)

S4 File Node feature vector of the Karate network. (CSV)
